# Response Inhibition and Binge Drinking During Transition to University: An fMRI Study

**DOI:** 10.3389/fpsyt.2020.00535

**Published:** 2020-06-09

**Authors:** Samuel Suárez-Suárez, Sonia Doallo, Jose Manuel Pérez-García, Montserrat Corral, Socorro Rodríguez Holguín, Fernando Cadaveira

**Affiliations:** Department of Clinical Psychology and Psychobiology, Universidade de Santiago de Compostela, Santiago de Compostela, Spain

**Keywords:** binge drinking, response inhibition, Go/NoGo, fMRI, alcohol-related stimuli

## Abstract

**Background:**

Binge Drinking (BD), a highly prevalent drinking pattern among youth, has been linked with anomalies in inhibitory control. However, it is still not well characterized whether the neural mechanisms involved in this process are compromised in binge drinkers (BDs). Furthermore, recent findings suggest that exerting inhibitory control to alcohol-related stimuli requires an increased effort in BDs, relative to controls, but the brain regions subserving these effects have also been scarcely investigated. Here we explored the impact of BD on the pattern of neural activity mediating response inhibition and its modulation by the motivational salience of stimuli (alcohol-related content).

**Methods:**

Sixty-seven (36 females) first-year university students, classified as BDs (n = 32) or controls (n = 35), underwent fMRI as they performed an alcohol-cued Go/NoGo task in which pictures of alcoholic or non-alcoholic beverages were presented as Go or NoGo stimuli.

**Results:**

During successful inhibition trials, BDs relative to controls showed greater activity in the bilateral inferior frontal gyrus (IFG), extending to the anterior insula, a brain region usually involved in response inhibition tasks, despite the lack of behavioral differences between groups. Moreover, BDs displayed increased activity in this region restricted to the right hemisphere when inhibiting a prepotent response to alcohol-related stimuli.

**Conclusions:**

The increased neural activity in the IFG/insula during response inhibition in BDs, in the absence of behavioral impairments, could reflect a compensatory mechanism. The findings suggest that response inhibition-related activity in the right IFG/insula is modulated by the motivational salience of stimuli and highlight the role of this brain region in suppressing responses to substance-associated cues.

## Introduction

Alcohol is by far the most used drug among youth in Western countries, as informed in epidemiological reports by the ESPAD (*European School Survey Project on Alcohol and Other Drugs*) (1) and the SAMSHA (*Substance Abuse and Mental Health Services Administration*) (2). The actual consumption rate of this substance entails significant health and economic costs (3, 4) and is one of the main causes of death among young people and adolescents (4). In this regard, multiple studies have indicated that the age of onset of drinking may be a determining factor in the development of future alcohol use disorders (AUD), illicit drug dependence and different problem drinking patterns (5–9). For example, Hingson et al. (8) informed that an early drinking onset significantly increases the probability to engage in binge drinking (BD). This pattern of consumption, characterized by the intake of large amounts of alcohol in a short period of time (leading to a blood alcohol concentration of at least 0.08 g/dl) [National Institute on Alcohol Abuse and Alcoholism (NIAAA), (10)], has been linked to neural and neuropsychological anomalies (11–13) and it could be considered as an initial step for developing alcohol use disorders (7, 14, 15). Moreover, evidence from animal (16) and human (17) studies about the vulnerability of the adolescent brain to the neurotoxic effects of alcohol highlights the impact of alcohol consumption on brain development. Of particular concern is the upsurge in alcohol consumption that takes place once the legally allowed age is exceeded (i.e. 18–21 years in most countries) but brain maturation is still under development (18, 19). In this regard, previous studies have demonstrated that brain regions known to support cognitive control, such as the prefrontal cortex, mature late (20, 21) and are particularly vulnerable to the neurotoxic effects of alcohol consumption (22, 23). University students have been specifically identified as a population of interest, mainly due to the escalation in alcohol drinking and increased rates of BD during transition to university (24, 25), placing them in a vulnerable position to develop future AUD (15).

Neuroscientific models of addictive behaviors have proposed that impairments of two related processes—response inhibition and salience attribution—may underlie the development of substance use disorders (26–29). In line with dual-process models, evidence of an imbalance between impaired response inhibition and an increased impact of the motivational properties of drug-related stimuli has been reported in alcohol-dependent patients [for a review, see (30)]; however, less is known about this potential imbalance in young binge drinkers (BDs) [for a theoretical framework, see (31)].

Response inhibition, usually defined as the ability to withhold or suppress a prepotent response (32), is considered a key mechanism to adjust behavior to meet environmental demands. Different meta-analyses have revealed the involvement of a predominantly right-lateralized fronto-parietal network, including the inferior parietal lobule (IPL), inferior frontal gyrus (IFG), middle frontal gyrus (MFG) and anterior insula, in successful inhibition of responses (33–38), and have underlined the importance of the right inferior frontal cortex (36, 37, 39).

An extensive body of work in alcohol dependence has shown alterations in inhibitory control at behavioral and neural levels [for a review, see (40); for a meta-analysis, see (41)]. Studies centered on young BDs have also provided evidence for the hazardous effects of this pattern of consumption on inhibitory control processes [for a review, see (42)], although its impact on the neural network subserving response inhibition is still not well characterized. In this regard, neuroimaging studies have reported an increased neural activity in BDs compared to controls during successful response inhibition trials [(43); see also (44)], as well as the recruitment of different brain regions during failed inhibitions (45), even in the absence of behavioral differences between the groups.

Regarding the enhanced salience attribution to drug-related stimuli in alcohol-dependent patients, a recent meta-analysis has revealed increased neural activity in brain regions implicated in incentive salience, reward processing and habit circuitry (e.g. dorsal striatum, prefrontal areas, anterior cingulate cortex and insula) (46). This study has also indicated the presence of differences between heavy and light drinkers in the activity of parietal and temporal regions (46). Regarding non-clinical BDs, fMRI studies that assessed alcohol cue reactivity and implicit positive associations towards alcohol cues reported similar results, showing greater neural activity in BDs in comparison with light drinkers in several incentive salience- and reward-related areas including, but not limited to, the anterior cingulate cortex, insula and dorsal striatum (47, 48). Furthermore, greater neural activity to alcohol-related pictures in some of these regions predicted increases in drinking and more alcohol- related problems in a group of college students who transitioned to heavy drinking during a year follow-up period (49).

In line with the findings mentioned above and the principles of dual-process models, one could expect to find greater response inhibition impairments to alcohol-associated stimuli in both individuals with AUD and BDs. Studies with alcohol-dependent patients offer behavioral and neuroimaging evidence that supports this hypothesis. A recent systematic review indicates that patients with AUD tend to show increased recruitment of the inhibitory control neural network (comprising dorsolateral and ventrolateral prefrontal cortex) and the salience network (anterior cingulate, insula and IPL) during alcohol-related processing while showing decreased engagement of relevant brain networks during non-drug-related processing (29). This potential imbalance has been, however, scarcely investigated in BDs, with the few published studies reporting, at a behavioral level, both the presence (50) and absence (51–55) of differences in the percentage of false alarms to alcohol-related stimuli, and with, to our knowledge, only one neuroimaging study trying to disentangle the subjacent neural mechanisms of these effects (56). In this work, Ames et al. (56), using a Go/NoGo task that required the inhibition of a prepotent response to alcohol images (NoGo stimuli), reported an increased neural activity in BDs, compared to controls, in regions involved in cognitive control (i.e. dorsolateral prefrontal cortex, anterior cingulate and anterior insula) during successful inhibition trials, in the absence of behavioral differences in the proportion of inhibitory errors. However, this study only included alcohol images as NoGo stimuli, and it is thus not possible to determine if the observed neural response pattern was specifically related to successful inhibition of response to alcohol-related stimuli or to a more general response inhibition process.

Here, we performed event-related functional magnetic resonance imaging (fMRI) in first-year university students during an alcohol-cued Go/NoGo task with a twofold aim: (1) to investigate the association between BD and potential anomalies in inhibitory control, and (2) to examine whether response inhibition processes are affected by the motivational salience of stimuli (i.e. alcohol- or non-alcohol-related content). Based on previous findings (43), we hypothesized, first, that BDs, compared to controls, would show an increased activation during successful inhibition trials (independently of the type of stimulus) in brain areas commonly identified as involved in response inhibition, such as the IFG, anterior insula, MFG or the IPL (33, 35), in the absence of behavioral differences between groups. Second, in line with previous studies (56) we expected the pattern of neural activity mediating response inhibition to be modulated by the stimulus motivational value, reflected in increased engagement, in BDs relative to controls, of the response inhibition neural network when withholding a response to alcohol-related stimuli. At a behavioral level, based on previous findings (51–56) we did not expect to find any significant differences between groups in the proportion of inhibitory errors.

## Materials and Methods

### Participants

Eighty-five first-year university students (18-19 years old) were selected to participate in the neuroimaging assessment within the framework of a broader research on consequences of BD among university students (for ERPs results, see 54). Initially, 2,998 first-year students from the University of Santiago de Compostela (Spain) completed a classroom questionnaire assessing alcohol and other substance consumption, as well as sociodemographic information. This questionnaire included the adapted version of the Alcohol Use Disorders Identification Test (AUDIT) (57, 58), the short version of the Nicotine Dependence Syndrome Scale (NDSS-S) (59, 60) and the Cannabis Abuse Screening Test (CAST) (61, 62). In order to identify the most suitable participants among the initial 2,998 questionnaires, the following preselection criteria were applied to the classroom questionnaire: i) provision of contact information (phone number and/or email); ii) 18–19 years old; and iii) non-consumption of illegal drugs (except cannabis). From the initial 2,998 questionnaires, a total of 516 subjects were identified to meet these criteria and showed interest in participating in the study. These participants completed a semi-structured interview in which quantity and frequency of alcohol use over the past 180 days were assessed *via* the Timeline Follow-Back calendar (TLFB) (63). Additionally, those subjects who reported cannabis consumption at some time throughout their lives during the classroom questionnaire completed the Cannabis TLFB to assess their cannabis consumption over the past 90 days. Participants were also interviewed about personal and family history of psychopathological disorders and completed the Spanish version of the Symptom Checklist-90-Revised (SCL-90-R) (64) to ensure they met inclusion/exclusion criteria. Exclusionary criteria included the following: chronic medical conditions that could affect neurocognitive functioning (diabetes, hypothyroidism, liver diseases, etc.), history of neurological disorders or brain injury, personal history of DSM-IV-TR Axis I and/or II diagnosed disorders, a score above 90th percentile in the Global Severity Index (GSI) or in two or more symptoms dimensions of the SCL-90-R, family history of major psychopathological disorders in first-degree relatives (clinically diagnosed by a professional), family history of alcoholism or substance use disorders (at least two first-degree relatives or three or more first- or second-degree relatives), AUDIT scores > 20, use of psychoactive medications, use of illegal drugs (except occasional consumption of cannabis) in the last 6 months, non-corrected sensory deficits and MRI contraindications. All participants gave written consent and received monetary compensation for their participation.

Volunteers were classified as binge drinkers (BDs) if they reported one BD episode at least once a month for the last six months, or as controls (CN) if they did not reach the alcohol consumption threshold for being considered BDs. Binge episodes were defined as the consumption of ≥ 50 g (female) or ≥ 70 g (male) of alcohol in one drinking occasion (i.e. an equivalent measure of the 4/5 standard drinks criteria reported in the NIAAA’s definition of BD) (10). Participants were instructed to abstain from consuming alcohol 24 h prior to the scan session.

Of the 85 subjects who met the inclusion criteria and completed the neuroimaging assessment, five participants were excluded from the analysis due to technical problems during image acquisition, seven participants were excluded due to excessive head movement during scanning (more than 3 mm/degrees of movement in any of the six directions), five were excluded for outlier behavioral data (more than 3 SD above or below the group mean) and, lastly, one was excluded due to the presence of an artefact in the functional images. Hence, the final sample included 67 right-handed participants, with 32 BDs (20 females) and 35 CN (16 females) (see **Table 1** for complete demographic and alcohol use data).

**Table 1:** Demographic and substance use characteristics of the final sample (mean ± SD).

	Controls	BDs
*n* (females)	35 (16)	32 (20)
Age	18.08 ± 0.28	18.22 ± 0.42
Caucasian (%)	100	100
Age of drinking onset***	16.29 ± 1.04	15.22 ± 1.24
Total AUDIT score ***	1.94 ± 2.52	10.28 ± 4.06
Number of BD episodes, past 6 months ***	0.91 ± 1.75	22.91 ± 12.26
Average # drinks per drinking occasion ***	1.96 ± 1.53	6.78 ± 1.98

***p ≤.001,

AUDIT, Alcohol Use Disorders Identification Test; BD episode: consumption of ≥ 5 (female) or ≥ 7 (male) Spanish standard drinks (10 g of alcohol) in 1 occasion.

### Behavioral Task

**Figure 1:**
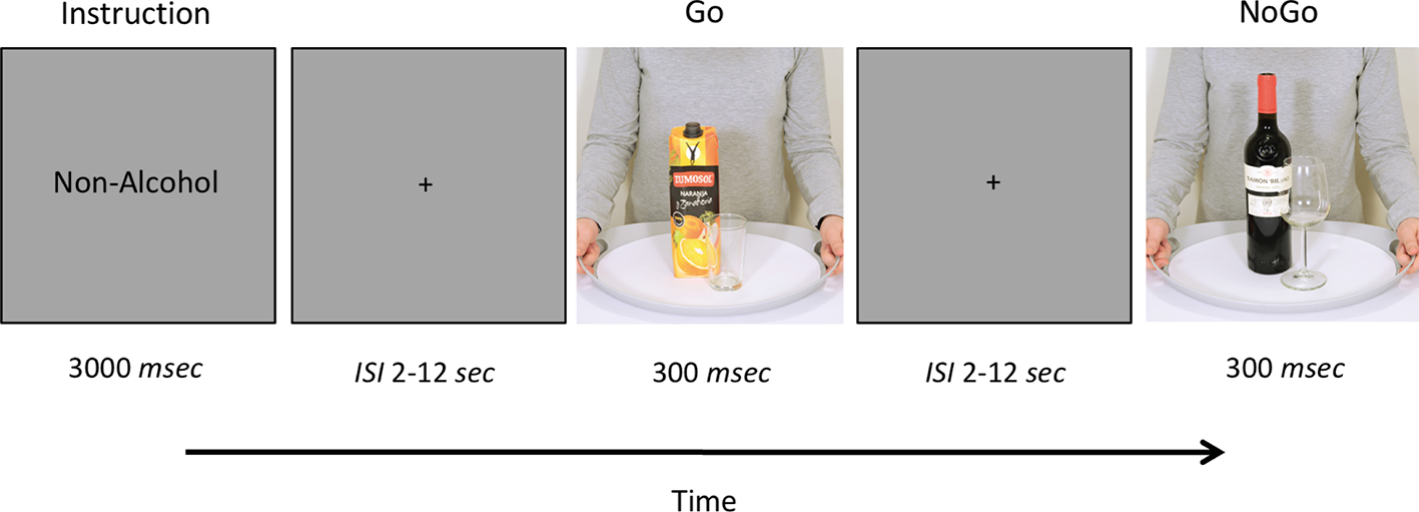
Schematic representation of the Go/NoGo task. Participants completed a Go/NoGo task with pictures of beverages (with or without alcoholic content) as stimuli. The task consisted in two blocks of 168 trials (25% NoGo trials). Before each block, participants were instructed to respond to one type of stimuli (Alcohol or Non-Alcohol) and to withhold their response to the other stimulus type.

During fMRI, participants completed a Go/NoGo task with pictures of alcoholic or non-alcoholic beverages as stimuli (see **Figure 1** for illustration). The picture set was designed to include drinks representative of Spanish consumption habits, comprising active pictures that display beverages being served, opened or consumed, following similar criteria to the Amsterdam Beverage Picture Set (ABPS) (65). Forty-eight pictures displaying different alcoholic beverages (beer, wine, and spirits) were used as alcohol-related stimuli, whereas 48 pictures displaying water, juice, dairy and soft drinks were used as non-alcoholic stimuli. All pictures had the same background and were scanned at a similar resolution and image size.

Stimuli were presented using the software Presentation (version 16.3, Neurobehavioral Systems Inc., Albany, CA; http://www.neurobs.com/) and were delivered through MRI-compatible video goggles (VisualSystem, NordicNeuroLab, Bergen, Norway), with a resolution of 800 x 600 pixels. The task included two blocks of 168 images each (126 Go trials and 42 NoGo trials). Each picture stimulus was presented on a light gray background for 300 ms at the center of the screen followed by a long and variable inter-stimulus interval (ISI) lasting 2–12 s, during which only a fixation cross was displayed. The durations for the ISIs were drawn from a logarithmic distribution that was skewed toward the shorter intervals (50% 2–4 s, 33% 4–8 s, 17% 8–12 s) (66). At the beginning of each block, participants were instructed to respond to one type of stimulus (Alcohol or Non-Alcohol), pressing a button with their right index finger on an MRI-compatible response grip (NordicNeuroLab, Bergen, Norway), as quickly as possible without making errors (Go trials), and to withhold their response to the other stimulus type (NoGo trials). Trial type (Go vs. NoGo) was randomized within each block and the order of blocks (Go Alcohol vs. Go Non-Alcohol) was counterbalanced across subjects.

Participants received a practice session of the task before they entered the scanner. Once in the scanner, and prior to each block, participants were informed, through a 3-s instructions screen, about the type of stimulus to which they must respond (i.e. Go Alcohol or Go Non-Alcohol trials).

### Behavioral Analysis

Reaction times (RTs) and the percentage of correct responses to Go stimuli, as well as the percentage of false alarms (FA) (i.e. response to NoGo stimuli), were submitted to 2x2x2 mixed-model analyses of variance (ANOVAs), with stimulus type (Alcohol, Non-Alcohol) as the within-subjects factor and group and gender as the between-subjects factors. Post-hoc comparisons were performed using the Bonferroni adjustment for multiple comparisons. All analyses were done with SPSS (version 21).

### fMRI Data Acquisition

Functional images were collected with a 3T Achieva Philips body scanner (Philips Medical Systems, Best, NL) equipped with a 32-channel SENSE head coil (located at the University Hospital Complex of Santiago de Compostela) using a T2*-weighted echo-planar imaging sequence with the following acquisition parameters: TR/TE = 3000/30 ms, flip angle = 87°, FOV = 230 × 230 mm, voxel size = 3 mm^3^, 45 axial slices. The task was conducted in one run consisting of about 700 volumes (~35 min). High-resolution anatomical T1-weighted images were also acquired using a 3D turbo field-echo sequence with the following parameters: TR/TE = 7.7/3.4 ms, flip angle = 8°, FOV = 240 mm, voxel size = 0.8 mm^3^, 200 transverse slices, acquisition time = 7 min.

### Image Processing and Analysis

Imaging data were processed and analyzed using Statistical Parametric Mapping (SPM8; http://www.fil.ion.ucl.ac.uk/spm/software/spm8/) implemented in Matlab (version 2015b, The Mathworks, Inc., Natick, MA). First, functional and anatomical images were reoriented to the anterior commissure. Then, functional images were corrected for slice timing and realigned and unwarped to correct for movement artefacts. The anatomical T1 images were coregistered to the realigned mean functional image, then images were transformed into standard MNI space using segmentation-based normalization parameters. The resulting functional images were spatially smoothed using a 7-mm FWHM Gaussian kernel. Blood oxygen level-dependent (BOLD) responses to each condition were modeled using an event-related design convolved with the canonical hemodynamic response function (HRF) to create regressors of interest (Go Alcohol, Go Non-Alcohol, NoGo Alcohol, NoGo Non-Alcohol). Instruction screens and response errors (i.e. failures to respond on Go trials or FA on NoGo trials) were modeled as effects of no interest. Additionally, movement parameters from the realignment step were included in the design matrix as regressors of no interest.

Individual t-contrasts were generated for each participant and then entered into a second-level random-effects analysis. Gender was included as a covariate in the analysis. We examined, first, the main effect for the response inhibition contrast (NoGo > Go) across all participants. The statistical threshold was set at p <.05 family-wise error (FWE) corrected for multiple comparisons at the voxel level across the whole brain.

Secondly, two-sample t-tests were defined to determine the between-group effects (i.e. BD > CN, BD < CN). Given our *a priori* hypotheses regarding differences in brain activity related to response inhibition (and its modulation by motivational salience), region of interest (ROI) analyses were conducted based on areas defined from a meta-analysis of Go/NoGo tasks involving complex stimulus identification (33) as follows: right IPL, right IFG, left IFG, right MFG, and right superior frontal gyrus (SFG) (see **Supplementary Table 1** for a list of ROIs coordinates). Contrasts were initially thresholded at p <.005 uncorrected and a cluster extent of 10 voxels; small volume correction (SVC) for multiple comparisons was then applied with a FWE-corrected threshold of p <.05 at cluster level within 10 mm-spheres centered on the reported coordinates after their transformation from Talairach into MNI space. To investigate the predicted higher BOLD activity in brain regions involved in response inhibition in BDs relative to CN, we examined the contrast NoGo > Go. Next, we examined modulation of NoGo vs. Go trial activation by the alcohol- vs. non-alcohol-related stimulus content. Our prediction of greater engagement in BDs (vs. CN) in areas mediating response inhibition when stimuli convey motivational salience was tested by a two-sample t-test comparing NoGo Alcohol > Go Non-Alcohol trials. To ensure that potential group differences in this contrast were directly linked to inhibiting responses to alcohol-related stimuli, and not simply due to general differences in inhibitory control processes, activation in NoGo Non-Alcohol > Go Alcohol trials was also explored. Finally, to test whether any observed activation differences between groups were due to overall differential reactivity to alcohol cues, we explored the contrast Alcohol > Non-Alcohol.

Additional Pearson’s correlation analyses were performed between parameter estimates extracted from each ROI showing significant between-group differences and: i) the age at drinking onset; ii) the number of BD episodes in the last 6 months, as a measure of the intensity of the BD pattern.

## Results

### Behavioral Performance

There were no significant differences between groups (BDs vs. CN), neither in the response to Go stimuli (i.e. percentage of hits or RTs) nor in the number of commission errors (i.e. percentage of FA) to NoGo stimuli. A significant main effect of stimulus type for the percentage of hits [**F**_(1,__63)_ = 28.288, p <.001] revealed greater accuracy for alcohol-related than for non-alcohol-related stimuli irrespective of the participant’s consumption pattern. There was also a significant gender by group interaction for the percentage of correct responses to Go stimuli [**F**_(1,__63)_ = 7.59, p = .008], which was explained by higher accuracy in males of the BD group (95.57 ± .96) relative to the CN group (92.88 ± .76) (p = .032), with no significant group differences in females (93.49 ± .74 vs. 95.36 ± .83; p = .098). Behavioral data are summarized in **Table 2**.

**Table 2:** Behavioral data for Control and BD groups (mean ± SD).

Behavioral Performance	Controls		Binge Drinkers	
	Alcohol	Non-Alcohol	Alcohol	Non-Alcohol
Reaction Time (Go trials, ms)	632 ± 107	642 ± 100	632 ± 92	616 ± 86
% Correct responses (Go trials)	96.98 ± 3.26	91.04 ± 5.88	96.53 ± 4.84	92.01 ± 6.11
% False alarms (NoGo trials)	6.39 ± 5.80	6.94 ± 6.69	7.07 ± 6.36	7.66 ± 6.27

### fMRI Results

**Figure 2:**
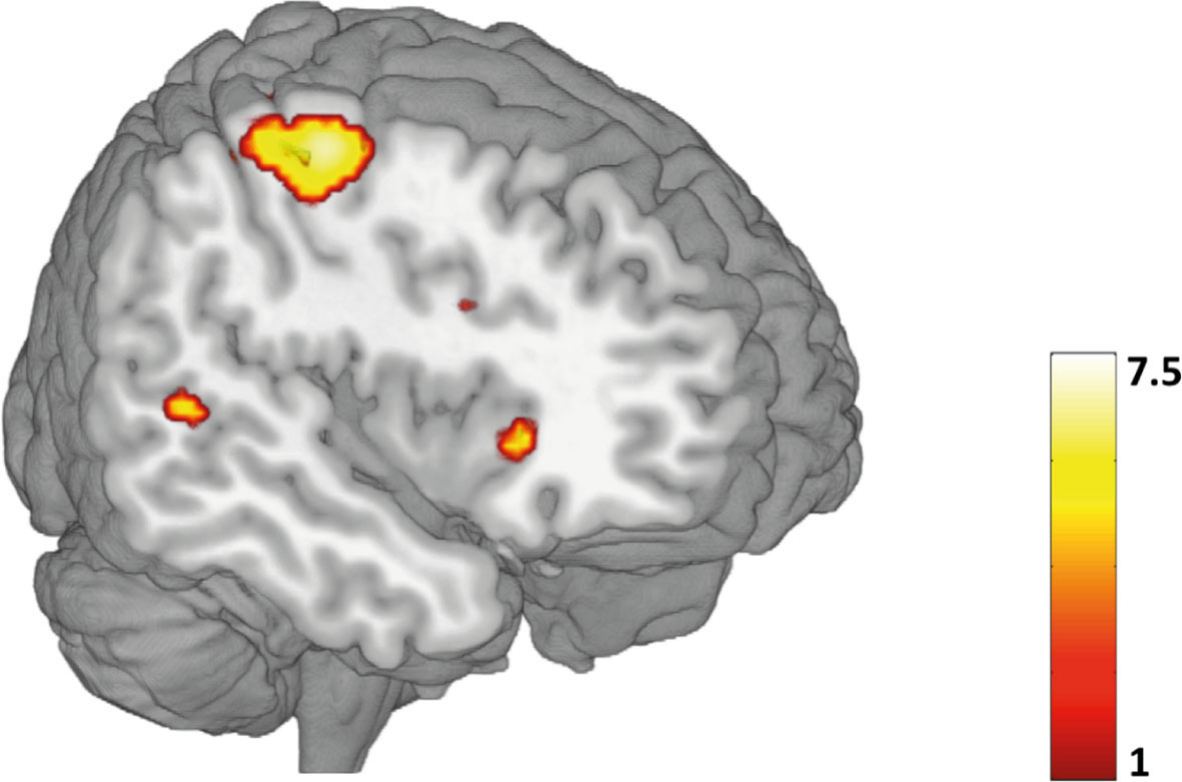
Whole-brain task activation during correct inhibition (NoGo > Go) for the whole sample (p <.05, FWE corrected). Color bar represents t-values. A list of all significant activations for this contrast can be found in **Table 3**.

Whole-brain analysis for the whole sample revealed significant BOLD activations during successful inhibition (NoGo > Go) in different areas of the right hemisphere including the precentral gyrus, IPL, IFG, MFG and SFG (see **Table 3**, **Figure 2**). These regions have been identified to be involved in Go/NoGo tasks in different meta-analysis (33–35). These results validate the ability of our task to tap into neural mechanisms related to response inhibition.

**Table 3:** Regions activated at whole-brain analysis in the contrast NoGo > Go for the whole sample.

Region (BA)	Side	x(mm)	y(mm)	z(mm)	k	*t*
Precentral/Postcentral Gyrus (4/3)	Right	40	-14	56	571	7.05
Precentral/Postcentral Gyrus (6)	Right	60	-10	38	108	6.74
Superior/Inferior Parietal Lobule (7)	Right	32	-54	52	237	6.59
Inferior Frontal Gyrus (47)	Right	30	28	-2	49	6.20
Middle Frontal Gyrus (9)	Right	42	20	26	45	5.73
Superior Temporal Gyrus (22)	Right	56	-38	4	19	5.55
Superior/Middle Frontal Gyrus (9)	Right	32	46	34	20	5.51
Superior Occipital Cortex/Precuneus (7)	Right	26	-84	28	33	5.47

BA, Brodmann Area.

All results are significant at p <.05 whole-brain voxel-level FWE corrected and cluster size (k) ≥ 10.

**Figure 3:**
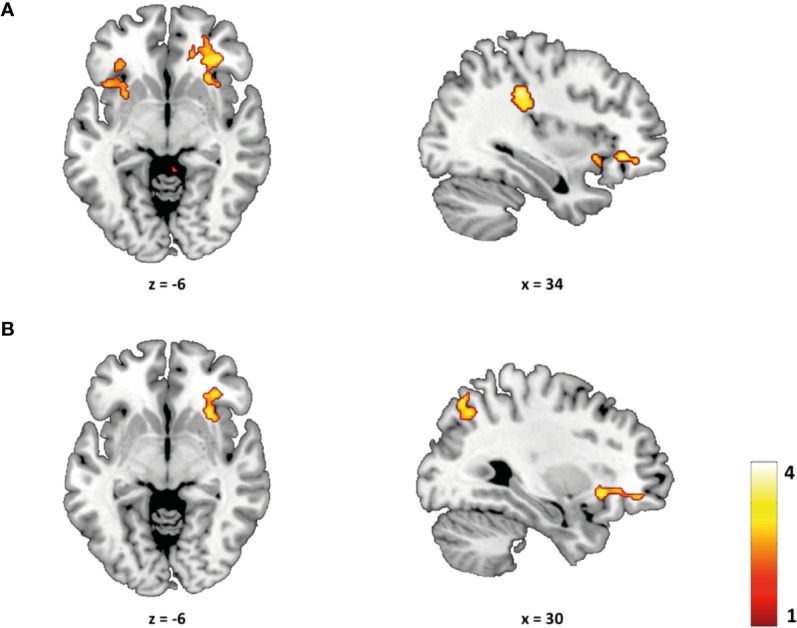
Group differences (BD > CN) during successful inhibition. **(A)** Compared to controls, BDs showed a greater BOLD activity in the NoGo vs. Go contrast in the bilateral inferior frontal gyrus, extending to the anterior insula (BA 47) (p <.05 FWE-small volume corrected). **(B)** BDs, in comparison with controls, showed a greater BOLD activity in the NoGo Alcohol vs. Go Non-Alcohol contrast in the right inferior frontal gyrus/anterior insula (BA 47) (p <.05 FWE-small volume corrected). T-maps are thresholded at p <.005 (uncorrected) and k ≥ 150 for display purposes only.

ROI analyses revealed significant differences between groups in BOLD response during successful response inhibition (NoGo > Go). Specifically, BDs showed greater activity, in comparison with CN, in the bilateral BA47 (IFG extending to the anterior insula) during NoGo relative to Go trials (**Table 4**, **Figure 3**). Furthermore, the NoGo Alcohol > Go Non-Alcohol contrast revealed a significant increased activation in this region (BA 47), restricted to the right hemisphere, in BDs relative to the CN group, when inhibiting a prepotent response to alcohol-related stimuli (**Table 4**, **Figure 3**). However, activations in the NoGo Non-Alcohol > Go Alcohol and Alcohol > Non-Alcohol contrasts did not reach statistical significance. This pattern of results suggests the modulation of the right IFG/insula activity to be specifically associated with suppressing responses to alcohol-related stimuli. The ROI analysis did not show any significant regions of increased activity in CN compared with BDs.

**Table 4:** Regions showing significant group differences (BD > CN) in BOLD response to successful inhibition.

Contrast	Region (BA)	Side	*P (*FWE)*	x(mm)	y(mm)	z(mm)	k	*t*
NoGo > Go	IFG/Insula (47)	Right	0.031	34	34	-6	74	3.64
	IFG/Insula (47)	Left	0.018	-30	16	-2	114	3.30
NoGo Alcohol > Go Non-Alcohol	IFG/Insula (47)	Right	0.031	30	20	-6	72	3.57

ROI analyses were based on the coordinates informed in the meta-analysis by Criaud and Boulinguez (33).

*Small volume correction (FWE, p <.05).

Correlation analysis performed in the BD group did not yield significant relationships (all p >.05) with any of the variables explored (i.e. age of onset of alcohol consumption, number of BD episodes in the last 6 months).

## Discussion

The main objective of this study was to investigate the link between BD and inhibitory control differences, with special consideration to the relationship between response inhibition and alcohol-related processing. We first analyzed the association between BD and behavioral performance in an alcohol-cued Go/NoGo task. We then explored the task-related neural correlates for both BDs and controls. Finally, we considered if there were differences in neural activity in BDs, relative to controls, when exerting inhibitory control to alcohol-related cues.

Consistent with most of the previous studies using Go/NoGo tasks in BDs (43, 67–72), we did not find behavioral differences related to BD in the examined response inhibition indices (i.e. FA) (irrespective of stimulus content). Furthermore, when alcohol cues were analyzed separately from non-alcohol cues, no significant differences in performance between BDs and CN were found. This finding is in line with a recent ERPs study from our group (54) and with previous studies reporting no differences in the number of FA (51–53, 55, 56), although some others have found an alcohol-cue-specific impairment of response inhibition (50).

In line with our hypothesis, the present neuroimaging results revealed an increased BOLD response in the bilateral IFG extending to the anterior insula (BA 47), in BDs compared to controls during successful inhibition trials, a region usually involved in response inhibition tasks (39, 73–75). As proposed by Aron and Poldrack (73), the inferior frontal cortex (IFC) modulates the interaction between the pre-supplementary motor area and the subthalamic nucleus (STN) within the neural network of response inhibition. Specifically, IFC sends excitatory impulses to the STN *via* the “hyperdirect” pathway and the STN sends excitatory output to the globus pallidus, which results in thalamus inhibition (76, 77). Looking at the characteristics of this neural circuitry, the increased activity in the IFG in the BD group could be interpreted as a greater recruitment of neural resources to successfully inhibit a response. Therefore, the differences observed in this region could be interpreted as part of a compensatory mechanism to attenuate the impact of abnormal brain activity on performance, in line with previous fMRI studies showing that BD is associated with increased neural activation, in the absence of behavioral impairments, during successful inhibition (43, 56).

It is worth noting that while BD tends to be associated with increased neural response, AUD has been associated with reduced response in fronto-parietal regions (e.g., IFG, MFG, IPL) (78, 79). Further exploring this relationship, Worhunsky and colleages (80) have reported two different activity patterns associated with the escalation of maximum number of drinks consumed in a single episode (MaxDrinks). First, escalating drinkers showed a hyper-engagement of fronto-parietal control mechanisms during successful relative to unsuccessful inhibition trials compared to constant (low) drinkers. On the other hand, when the group of escalating drinkers was divided according to their MaxDrinks scores, a greater MaxDrinks was associated with reduced engagement of the fronto-parietal network. These findings thus suggest a transition, in terms of neural activation, from an initial consumption stage (related to hyperactivation) to a more problematic drinking (related to reduced engagement of neural resources).

Consistent with our second hypothesis and the findings reported by Ames et al. (56), we observed a greater activity in BDs (vs. controls) during response inhibition to alcohol-related stimuli. Specifically, BDs showed greater activity in the right IFG/insula (BA 47) when inhibiting a response to alcohol stimuli (NoGo Alcohol > Go Non-Alcohol). However, no significant differences were observed when participants were asked to inhibit their response to non-alcohol stimuli (NoGo Non-Alcohol > Go Alcohol). These findings suggest that response inhibition-related activity in the right IFG/insula seems to be modulated by the motivational salience of stimuli and highlight the role of this region in suppressing responses to substance-related cues. Also, these results are in agreement with previous studies, in the general population, showing that response inhibition could be modulated by the motivational content of stimuli (81–85). In particular, some of these works using fMRI to characterize the neural basis of this modulation reported increased neural activity of the IFG/insula in trials where a response to reward-related stimuli had to be inhibited (82, 85). It should be taking into account that the IFG and the anterior insula, besides being considered key regions for response inhibition, have been also proposed to be part of the salience network (82, 86, 87) and, therefore, they may be involved in adjusting cognitive control to motivational demands of the context (86). In this regard, a meta-analysis has suggested that the right anterior insula would play an important role in detection of behaviorally relevant salient events, whereas the right IFG would be more involved in exerting inhibitory control (36). Therefore, it is possible that the higher activity observed in BDs in the right BA 47 is pointing to the presence of differences associated with the alcohol consumption pattern in the interaction between cognitive control and salience detection processes.

The current research extends prior results about BD-related anomalies in inhibitory control mechanisms and provides new evidence about increased IFG/insula activity, a key region for inhibitory control, during response inhibition to alcohol-related stimuli. Some limitations of the present study should be noted. First, the lack of assessment before participants engaged in BD prevents us from establishing potential pre-existing differences between groups that may explain the observed results, as indicated by previous investigations (43, 88, 89). A future follow-up will allow us to deepen the relationship between BD pattern and neural anomalies in frontal regions and to explore if these early neural differences may subtend the transition from recreational to pathological consumption patterns. Second, in contrast with how Go/NoGo tasks are typically designed to be performed in behavioral experiments, the characteristics of the hemodynamic response led us to employ a relatively slow and unpredictable stimulus presentation which could reduce the prepotent tendency to respond. Therefore, this type of approach may be less sensitive for assessing behavioral differences and it should be taken into account when explaining the lack of differences between the groups. Third, similarly to previous studies [e.g. (56, 90)], our task involves a much more demanding cognitive context than classic Go/NoGo tasks, with attention, stimulus categorization and response selection processes being highly intertwined, so research findings should be interpreted with this limitation in mind. One final limitation should be noted: neither individual preferences nor potential differences in familiarity of the employed images were addressed in the present study.

In summary, our results revealed that young BDs showed increased frontal activity, relative to controls, during successful inhibition trials in an alcohol-cued Go/NoGo task, despite a lack of behavioral differences between groups. These findings provide new evidence about the role of the IFG, extending to the anterior insula, as an important region to explore neural differences associated with BD, as well as suggest a specific involvement of this region in withholding a prepotent response to stimuli with alcohol-related content. At a more clinical level, our study provides subclinical information in a healthy population, without evidence of behavioral problems, which highlights the risk of this form of consumption. In addition, this investigation emphasizes the relevance of assessing the cognitive processes of interest using specific substance-related cues to better understand the relationship between those processes and the alcohol consumption pattern, and provides useful information to contribute to the development of future prevention strategies as suggested by studies focused on inhibitory control training and cognitive bias modification (see 91 for an insightful review of neuroscience findings and treatment programs).

## Data Availability Statement

The datasets generated for this study are available on request to the corresponding author.

## Ethics Statement

The studies involving human participants were reviewed and approved by Bioethics Committee of the Universidade de Santiago de Compostela. The patients/participants provided their written informed consent to participate in this study.

## Author Contributions

FC, SD, SR, and MC designed the study. SS-S and JP-G participated in the data collection. MC was responsible for sample selection. SD and SR designed the experimental task. SS-S analyzed the data. FC, SD, and SS-S interpreted the data. SS-S wrote the article. All authors contributed to the article and approved the submitted version.

## Funding

This investigation was supported by grants from the Spanish Ministerio de Sanidad, Servicios Sociales e Igualdad, Plan Nacional sobre Drogas (PNSD 2015/034), Ministerio de Economía y Competitividad (PSI2015-70525-P) co-funded for European Regional Development Fund and Xunta de Galicia (GRC ED431C 2017/06). SS-S was supported by a predoctoral fellowship from the Spanish Ministerio de Economía y Competitividad (BES-2016-076298). JP-G was supported by the FPU program from the Spanish Ministerio de Educación, Cultura y Deporte (FPU16/01573).

## Conflict of Interest

The authors declare that the research was conducted in the absence of any commercial or financial relationships that could be construed as a potential conflict of interest.
