# Supplementary material for: Response Inhibition and Binge Drinking During Transition to University: An fMRI Study
Source: Front Psychiatry. 2020 Jun 9;11:535. doi: 10.3389/fpsyt.2020.00535 (PMC7296115; doi:10.3389/fpsyt.2020.00535)
Supplement: Supplementary file 1 [file Table_1.docx]

Supplementary Material for The Article:

Response Inhibition and binge drinking during transition to university: an fMRI study

Samuel Suárez-Suárez^*^, Sonia Doallo, Jose Manuel Pérez-García, Montserrat Corral, Socorro Rodríguez Holguín, Fernando Cadaveira

Department of Clinical Psychology and Psychobiology, Universidade de Santiago de Compostela, Santiago de Compostela, Spain

*** Correspondence:**Samuel Suárez-Suárez

Department of Clinical Psychology and Psychobiology

Universidade de Santiago de Compostela

Campus Vida, s/n, 15782 Santiago de Compostela

Galicia-Spain
Email: [samuel.suarez@usc.es](mailto:samuel.suarez@usc.es)

***Supplementary Table 1.*** List of coordinates of the regions of interest and the peak activations reported in the meta-analysis by Criaud and Boulinguez (2013).

| Cluster | Region (BA) | Side | Cited activation in Talairach coordinates (x, y, z) | ROI center in MNI coordinates (x, y, z) |
| --- | --- | --- | --- | --- |
| 1 | IPL (40) | Right | 43, -48, 42 | 42, -52, 45 |
| 2 | IFG (47) | Right | 35, 22, -3 | 37, 26, -8 |
| 3 | IFG (47) | Left | -33, 18, -3 | -34, 22, -7 |
| 4 | MFG (9) | Right | 35, 41, 28 | 36, 41, 29 |
| 5 | SFG (6) | Right | 9, 6, 55 | 9, 2, 61 |

BA: Brodmann area; IPL: inferior parietal lobule; IFG: inferior frontal gyrus; MFG: middle frontal gyrus; SFG: superior frontal gyrus; ROI: Region of interest
